# Genkwanin Inhibits Proinflammatory Mediators Mainly through the Regulation of miR-101/MKP-1/MAPK Pathway in LPS-Activated Macrophages

**DOI:** 10.1371/journal.pone.0096741

**Published:** 2014-05-06

**Authors:** Yuan Gao, Fen Liu, Lei Fang, Runlan Cai, Chuanjie Zong, Yun Qi

**Affiliations:** Institute of Medicinal Plant Development, Chinese Academy of Medical Sciences & Peking Union Medical College, Beijing, China; University of Barcelona, Spain

## Abstract

Genkwanin is one of the major non-glycosylated flavonoids in many herbs with anti-inflammatory activities. Although its anti-inflammatory activity *in vivo* has been reported, the potential molecular mechanisms remain obscure. In this study, by pharmacological and genetic approaches, we explore the anti-inflammatory effects of genkwanin in LPS-activated RAW264.7 macrophages. Genkwanin potently decreases the proinflammatory mediators, such as iNOS, TNF-α, IL-1β and IL-6, at the transcriptional and translational levels without cytotoxicity, indicating the excellent anti-inflammatory potency of genkwanin *in vitro*. Mechanism study shows that genkwanin significantly suppresses the p38- and JNK-mediated AP-1 signaling pathway and increases the mitogen-activated protein kinase (MAPK) phosphatase 1 (MKP-1) expression at the posttranscriptional level. We also confirmed that microRNA-101 (miR-101) is a negative regulator of MKP-1 expression. Moreover, regardless of miR-101-deficient cells or miR-101-abundant cells, the suppression effects of genkwanin on supernatant proinflammatory mediators' levels are far less than that in respective negative control cells, suggesting that genkwanin exerts anti-inflammatory effect mainly through reducing miR-101 production. However, genkwanin can't affect the level of phospho-Akt (p-Akt), indicating that the phosphorylation of Akt may be not responsible for the effect of genkwanin on miR-101 production. We conclude that genkwanin exerts its anti-inflammatory effect mainly through the regulation of the miR-101/MKP-1/MAPK pathway.

## Introduction

Genkwanin is one of the major non-glycosylated flavonoids in some herbs which have anti-inflammatory activities, such as Genkwa Flos (*Daphne Genkwa* Sieb. et Zucc.) [1], rosemary (*Rosmarinus officinalis* L.) [2] and the leaves of *Cistus laurifolius* L. [3]. Previous pharmacological studies have found that genkwanin has a variety of pharmacological effects including anti-bacterial [4], [5], antiplasmodial [6], radical scavenging [7], chemopreventive [8] and inhibiting 17β-Hydroxysteroid dehydrogenase type 1 [9] activities. Although Pelzer *et al*. [10] has reported that genkwanin could inhibit the development of cotton-pellet-induced granuloma in rat, the potential molecular mechanisms of the anti-inflammatory activity of genkwanin remain obscure.

Inflammation is a central feature of many pathophysiological conditions in response to tissue injury and host defenses against invading microbes [11]. Key events in the inflammatory process include the expression of inflammatory cytokines, chemokines, and other mediators [12]. Macrophages play a central role in host defense against pathogen microbes by recognizing bacterial components and resulting in the activation of an arsenal of anti-microbiol effectors and initiation of the inflammatory cascade [13], [14]. LPS, a major component of the outer membranes in Gram-negative bacteria, can be recognized by a TLR4 receptor complex [15]. Stimulation of TLR4 by LPS triggers the recruitment of adaptor protein MyD88, which in turn transmits a series of signaling cascades that lead to the activation of mitogen-activated protein kinase (MAPK) [16]-[19]. The MAPK is a group of highly conserved serine/threonine protein kinases, including p38, ERK1/2 and JNK. Once activated, MAPK phosphorylate downstream protein kinases and transcription factors, leading to the production of proinflammatory cytokines, such as iNOS, TNF-α, IL-1β and IL-6, etc.

The objective of the present paper is to clarify the anti-inflammatory mechanisms of genkwanin in LPS-activated RAW264.7 macrophages. Our results indicate that genkwanin suppresses the production of inflammatory mediator in LPS-activated RAW264.7 macrophages mainly through mediating microRNA-101 (miR-101)/MAPK phosphatase 1 (MKP-1)/MAPK pathway.

## Materials and Methods

### Materials

The murine macrophage RAW264.7 cell line was purchased from American Type Culture Collection (ATCC, Rockville, MD, USA). Genkwanin (≥98%) was purchased from Rochen Co. (Shanghai, China) and dissolved in DMSO at the concentration of 10 mg/mL. We confirmed that genkwanin from other source, the National Institutes for Food and Drug Control (Beijing, China), exhibited equivalent effects in crucial experiments. Mouse TNF-α and IL-6 ELISA kits were obtained from Biolegend Co. (San Diego, California, USA). Mouse IL-1β ELISA kit was obtained from Excell Technology Co. (Shanghai, China). Antibodies for iNOS, MKP-1 and β-actin were obtained from Santa Cruz Biotechnology, Inc. (Santa Cruz, California, USA). Antibodies for MAPK and Akt were from Cell Signaling Technology (Danvers, Colorado, USA). The plasmids for NFκB-TA-luc, AP1-TA-luc and their controls GL6-TA were from Beyotime Institute of Biotechnology (haimen, Jiangsu, China). The luciferase assay system and lipofectamine™ 2000 reagent were purchased from Promega Co. (Madison, Tennessee, USA) and Invitrogen (New York, California, USA), respectively. Horseradish peroxidase-conjugated anti-rabbit or mouse IgG secondary antibodies were obtained from Jackson ImmunoResearch Laboratories, Inc. (Lancaster, Philadelphia, USA). miR-101 qPCR kit with U6 snRNA (control), dsRNA mimic and ssRNA inhibitor for mmu-miR-101a were obtained from Genepharma (Shanghai, China). All other reagents were of analytical grade.

### Cell culture and treatment

RAW264.7 macrophages were grown in DMEM supplemented with 10% heat-inactivated FBS and 1.0% penicillin-streptomycin solution in a humidified incubator with 5.0% CO_2_ at 37°C. When performing genkwanin treatment, genkwanin was first added to the culture medium and then mixed thoroughly (the final concentrations of DMSO were ≤0.15%). The cell culture medium was replaced with the medium already mixed with genkwanin.

### Measurement of nitrite

Cells were pretreated with genkwanin at the indicated concentrations for 2 h and then exposed to LPS (10 ng/mL) for 24 h. The nitrite concentration in the medium was measured as an indicator of NO production according to the Griess reaction. 100 µL of each supernatant was mixed with the same volume of Griess reagent (1% sulfanilamide in 5% phosphoric acid and 0.1% N-1-naphthylethylenediamine dihydrochloride in water). The absorbance was measured at a wavelength of 540 nm after incubation for 10 min. The nitrite concentration was calculated with reference to a standard curve of sodium nitrite generated from known concentrations. L-NAME was used as a positive control.

### Measurement of iNOS enzyme activity

The activity of iNOS was assayed as previously described [20] with slight modifications. The cells were plated in a 25 cm^2^ culture flask and incubated with LPS (Sigma, Escherichia coli 055: B5; 10 ng/mL) for 12 h. After being washed twice by PBS, the cells were harvested and plated in a 48-well plate, and incubated in the presence or absence of genkwanin at different concentrations for a further 12 h. The iNOS activity was assayed by measuring the nitrite level in the supernatant by Griess method. L-NAME was used as a positive control.

### ELISA for TNF-α, IL-1β and IL-6

For the measurements of TNF-α, IL-6 and IL-1β, RAW264.7 macrophages were pretreated with genkwanin for 2 h and then stimulated with LPS (10 ng/mL) for 24 h. TNF-α, IL-6 and IL-1β in the cell supernatants were assayed using ELISA kits according to the manufacturer's instructions. The concentrations were calculated from the standard curves.

### Quantitative real-time PCR (RT-qPCR)

The cells were pretreated with genkwanin at the indicated concentrations for 2 h and then exposed to LPS (10 ng/mL) for 4 h. The RNA extraction and RT-*q*PCR assays for the mRNA levels of iNOS, TNF-α, IL-1β and IL-6 were performed as we previously described [21]. RT-*q*PCR assay for the miR-101 level was performed according to the manufacturer's protocol. The cycling conditions were as follows: 95°C for 3 min, and then 40 cycles of 95°C for 12 s, 62°C for 40 s. The levels of iNOS, TNF-α, IL-1β and IL-6 were normalized to β-actin. The level of miR-101 was normalized to U6 snRNA.

### Transfection of plasmids, dsRNA mimic and ssRNA inhibitor for mmu-miR-101a

The sense strand and the antisense strand of dsRNA mimic for mmu-miR-101a were 5′-UAC AGU ACU GUG AUA ACU GAA-3′ and 5′-CAG UUA UCA CAG UAC UGU AUU-3′, respectively. The strand of ssRNA inhibitor against mmu-miR-101a was 5′-UUC AGU UAU CAC AGU ACU GUA-3′. Plasmids for pNFκB-TA-luc, pAP1-TA-luc and their controls pGL6-TA, and RNAs were transfected into RAW264.7 macrophages using lipofectamine™ 2000 as described in the manufacturer's protocol.

### Luciferase reporter assay

RAW264.7 macrophages were transfected with the luciferase reporter pAP-1-TA-luc (A) and pNF-κB-TA-luc (B). 24 h after transfection, the cells were pretreated with different concentrations of genkwanin for 2 h and then exposed to LPS (10 ng/mL). After 6 h, the cells were lysed and luciferase activity was measured using the Luciferase Assay System.

### Western blot analysis

RAW264.7 macrophages were pretreated with genkwanin at indicated concentrations for 2 h and then exposed to LPS (10 ng/mL) for 1 h (for p-p38, p-JNK, p-ERK1/2, MKP-1 and p-Akt assays) or 24 h (for iNOS assay). The western blot assay was performed as we previously described [21]. Proteins in nucleus or cytoplasm were extracted and separated by SDS-PAGE and transferred to polyvinylidene difluoride membranes. The membranes were blocked at room temperature for 4 h with 5.0% nonfat dry milk, and then incubated with each primary antibody at 4°C overnight. After washing, the membranes were incubated with HRP-conjugated secondary antibodies for 2 h at room temperature. The blots were visualized using enhanced chemiluminescence, and data were analysed using the Gel Doc EQ System (Bio-Rad).

### Statistical analysis

Data represent the mean ± SD of at least three independent experiments, each experiment was performed in triplicate. One-way ANOVA was used to determine the statistical significance between different groups. A student *t*-test was used when only two groups were compared. Differences were considered to be significant at *p* <0.05.

## Results

### Genkwanin inhibits LPS-induced NO production and suppresses iNOS at the transcriptional and translational levels

**Figure 1:**
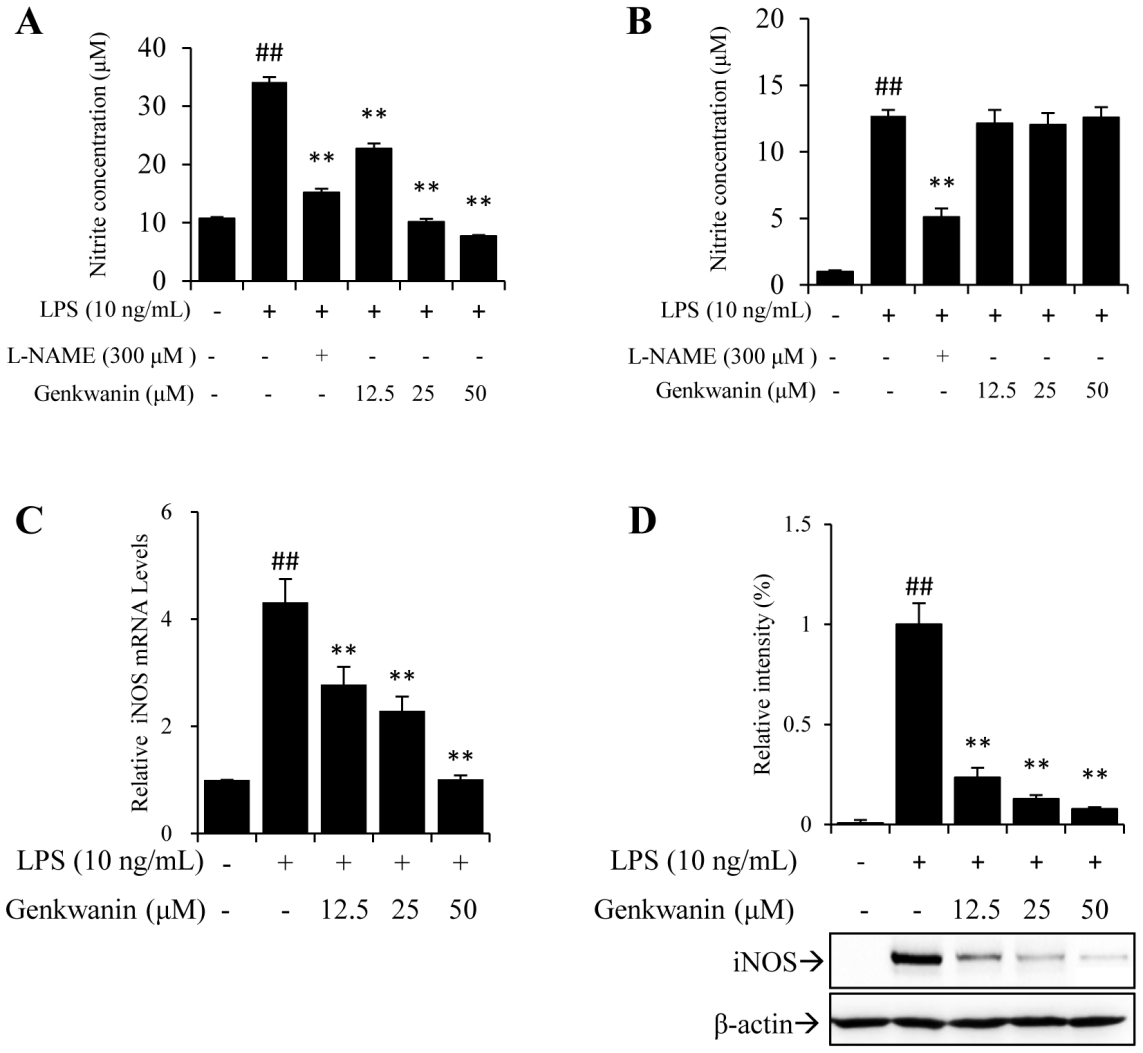
Effects of genkwanin on NO production and iNOS in LPS-activated RAW264.7 macrophages. (A) Effects of genkwanin on LPS-induced NO production. Cells were pretreated with genkwanin at the indicated concentrations for 2 h and then exposed to LPS (10 ng/mL) for 24 h. After treatment, nitrite levels in the medium were measured by Griess reaction. L-NAME was used as a positive control. (B) Effects of genkwanin on iNOS enzyme activity. Cells were pretreated with LPS (10 ng/mL) for 12 h and then exposed to genkwanin at the indicated concentrations for a further 12 h without LPS. Nitrite levels in the medium were measured. L-NAME was used as a positive control. (C) Effects of genkwanin on iNOS mRNA expression. Cells were pretreated with the indicated concentrations of genkwanin for 2 h and then exposed to LPS (10 ng/mL) for 4 h. mRNA of iNOS was determined by RT-*q*PCR analysis. (D) Effects of genkwanin on iNOS protein levels. Cells were pretreated with genkwanin at the indicated concentrations for 2 h and then exposed to LPS (10 ng/mL) for 24 h. After treatment, cellular proteins were prepared and the iNOS protein levels were determined by Western blot analysis. Bars represent mean ±SD of three independent experiments. ^##^ *p*<0.01 vs. normal control group; **p<0.01 vs. LPS alone.

Cell viability analysis showed that genkwanin did not affect the cell viability up to a concentration of 50 µM (Figure S1). NO, a small diffusible molecular generated by iNOS in activated macrophages, is closely related to many inflammatory diseases [22]–[25]. Thus, to investigate the effect of genkwanin on inflammation, we first measured supernatant NO production in LPS-stimulated RAW264.7 macrophages. As shown in Figure 1A, genkwanin inhibited the LPS-induced production of NO in a concentration-dependent manner. As we known, iNOS only expresses in the present of external stimulus [26]. To assay the effect of genkwanin on iNOS enzyme activity, we pretreated the cells with LPS for 12 h and then removed LPS. In a 48-well plate, the re-plated cells could not produce new iNOS in the absent of LPS. Under this condition, all change of NO production was attributed to the change of iNOS enzyme activity rather than iNOS mass. As shown in Figure 1B, genkwanin could not significantly affect the activity of iNOS.

Thus, we next investigated the inhibitory effects of genkwanin on iNOS mRNA and protein levels. As shown in Figure 1C–D, LPS stimulation of RAW264.7 macrophages resulted in a dramatic increase in iNOS at the transcriptional (Figure 1C) and translational (Figure 1D) levels. Treatment with genkwanin concentration-dependently inhibited the LPS-induced increase in iNOS mRNA expression and protein levels.

### Genkwanin suppresses LPS-induced TNF-α, IL-1β and IL-6 at the transcriptional and translational levels

**Figure 2:**
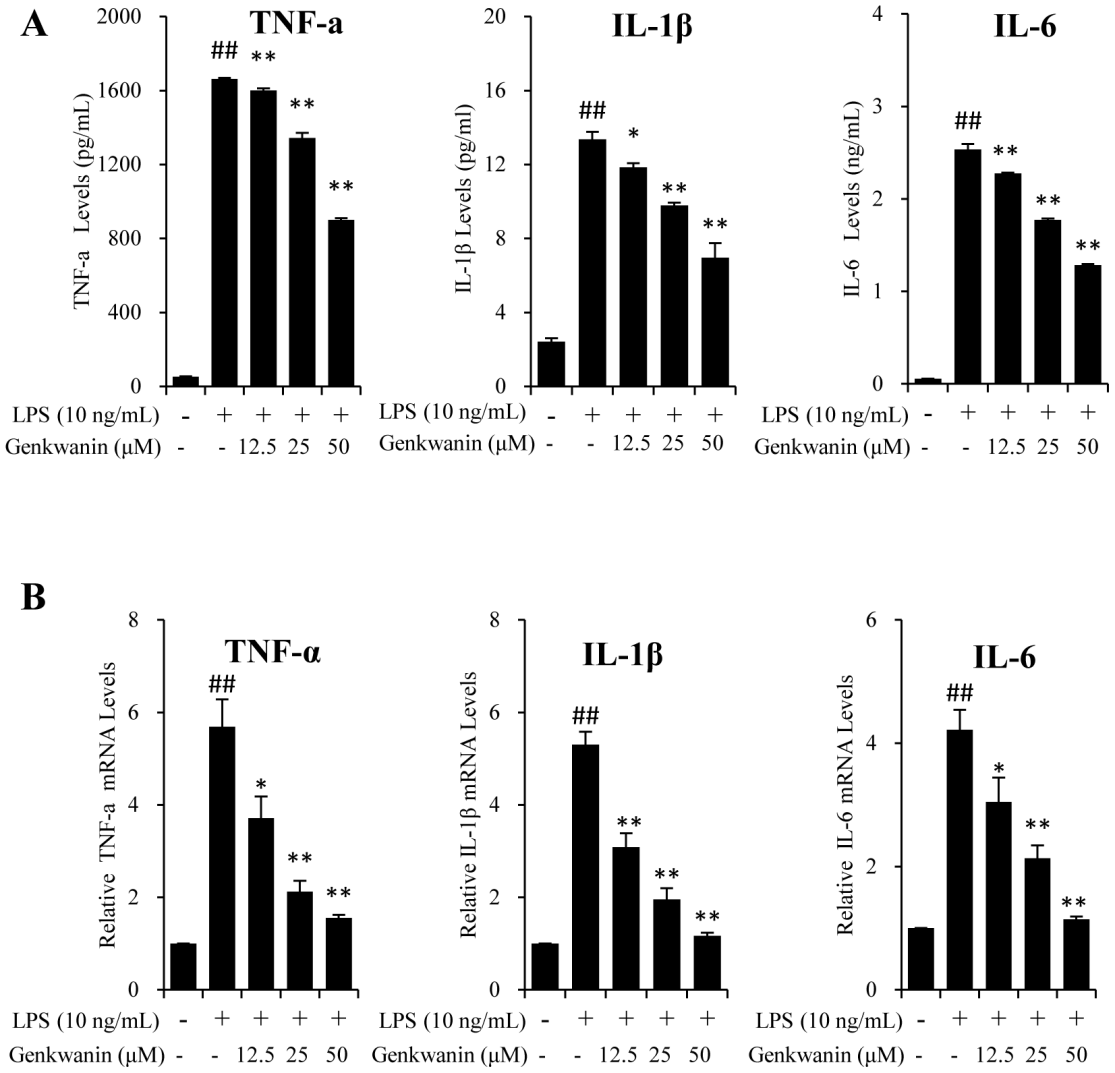
Effects of genkwanin on TNF-α,IL-1β and IL-6 in LPS-activated RAW264.7 macrophages at the transcriptional and translational levels. (A) The cells were pretreated with the indicated concentrations of genkwanin for 2 h and then exposed to LPS (10 ng/mL) for 24 h. The levels of TNF-α, IL-1β and IL-6 in the supernatant were determined by ELISA. (B) The cells were pretreated with genkwanin at the indicated concentrations for 2 h and then exposed to LPS (10 ng/mL) for 4 h. The mRNA expressions of TNF-α, IL-6 and IL-1β were determined by RT-*q*PCR analysis. ^##^ *p*<0.01 vs. normal control group; **p<0.01 vs. LPS alone. Bars represent mean ±SD of three independent experiments.

The effect of genkwanin on the production of proinflammatory cytokines was examined. As shown in Figure 2A, genkwanin suppressed the productions of TNF-α, IL-1β and IL-6 in LPS-stimulated RAW264.7 macrophages in a concentration-dependent manner. We next analysed the effects of genkwanin on the mRNA quantities of TNF-α, IL-1β and IL-6 by RT-*q*PCR. As shown in Figure 2B, genkwanin consistently down-regulated the LPS-induced transcription of TNF-α, IL-1β and IL-6 mRNA in a concentration-dependent manner.

### Genkwanin suppresses the LPS-induced phosphorylation of p38 and JNK via the up-regulation of MKP-1 expression

**Figure 3:**
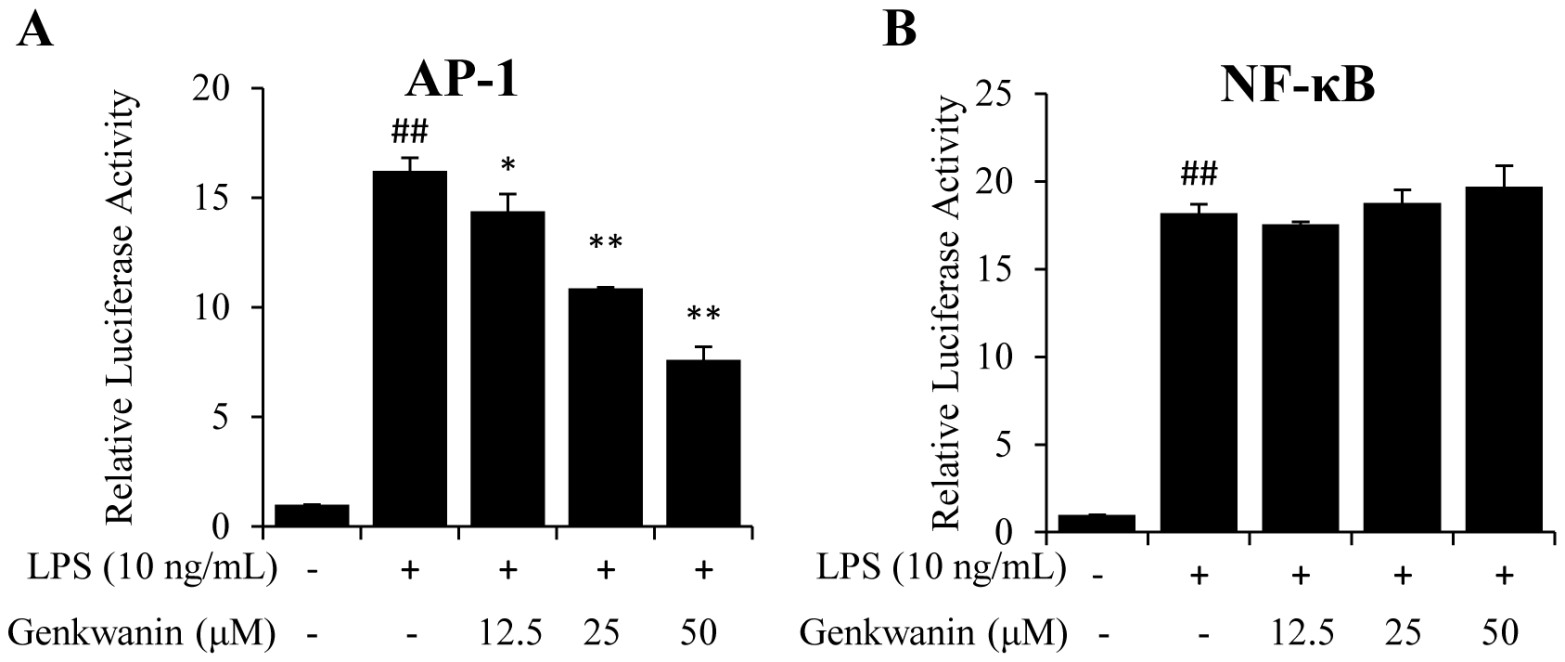
Effects of genkwanin on LPS-induced AP-1 and NF-κB activities. RAW264.7 macrophages were transfected with the luciferase reporter pAP-1-TA-luc (A) and pNF-κB-TA-luc (B). 24 h after transfection, the cells were pretreated with genkwanin for 2 h and then exposed to LPS (10 ng/mL) at the indicated concentrations. After 6 h, the luciferase activity was determined. ^##^ *p*<0.01 vs. normal control group; **p<0.01 vs. LPS alone. Bars represent mean ±SD of three independent experiments.

**Figure 4:**
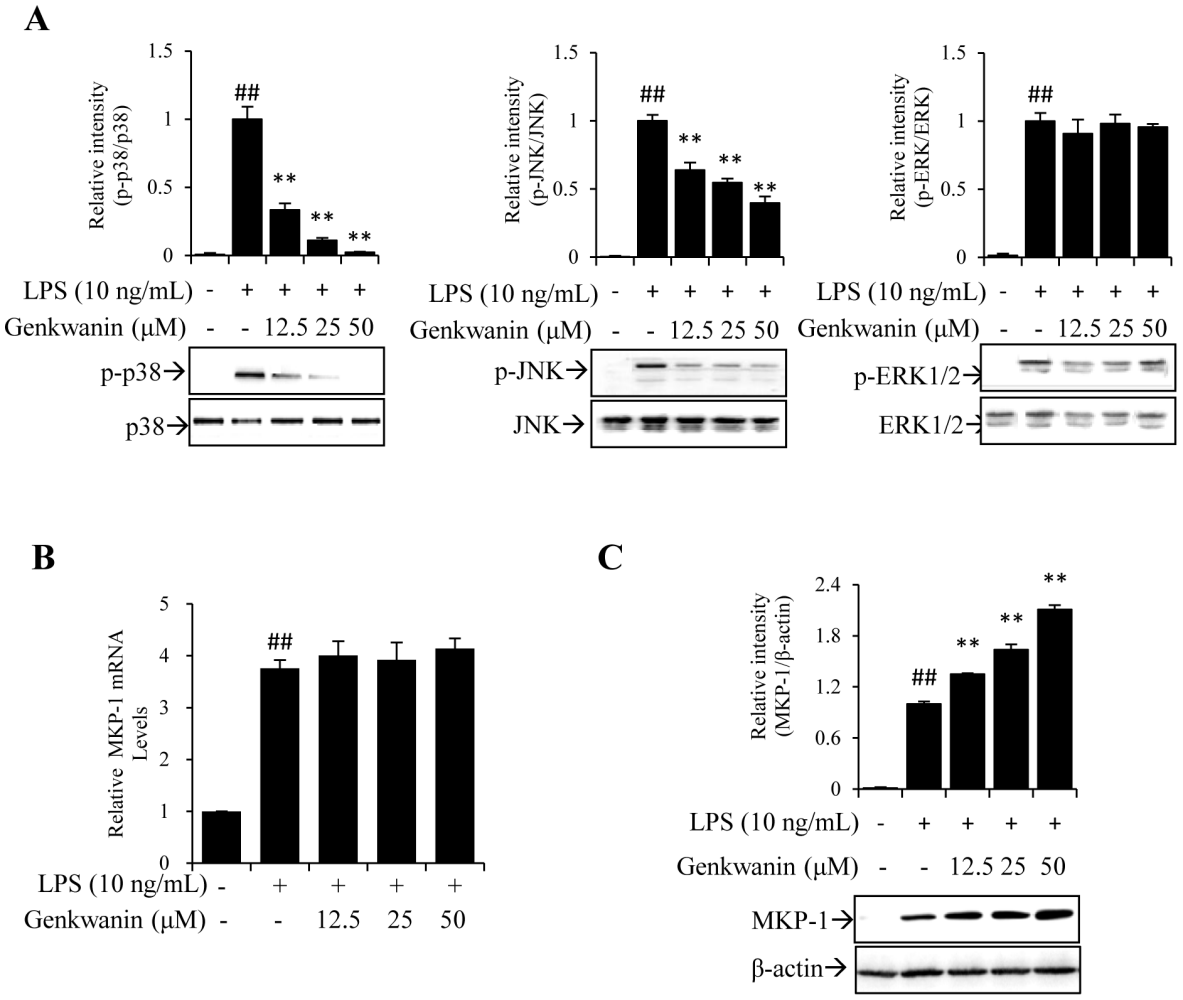
Effects of genkwanin on LPS-induced MAPK and MKP-1. (A) Effects of genkwanin on the LPS-induced phosphorylation of p38, JNK and ERK1/2. RAW264.7 macrophages were pretreated with genkwanin at indicated concentrations for 2 h and then exposed to LPS (10 ng/mL) for 1 h. Total proteins were extracted for the Western blot analysis. (B) Effects of genkwanin on MKP-1 mRNA in LPS-activated RAW264.7 macrophages. The cells were pretreated with genkwanin at the indicated concentrations for 2 h and then exposed to LPS (10 ng/mL) for 1 h. The mRNA expression of MKP-1 was determined by RT-*q*PCR analysis. (C) Effects of genkwanin on MKP-1 protein level in LPS-activated RAW264.7 macrophages. Cells were pretreated with genkwanin at indicated concentrations for 2 h and then exposed to LPS (10 ng/mL) for 1 h. Cell lysates were analysed by Western blot. ^##^ *p*<0.01 vs. normal control group; **p<0.01 vs. LPS alone. Bars represent mean ±SD of three independent experiments.

Since the induction of proinflammatory mediators by LPS is known to be predominantly regulated by NF-κB and AP-1 [27]–[30], we investigated if genkwanin exerts anti-inflammatory activities by affecting these two pathways. As shown in Figure 3, genkwanin significantly suppressed the AP-1 signaling pathway (Figure 3A) but had little effect on the NF-κB signaling pathway (Figure 3B). Thus, we next explored the MAPK, which mainly act upstream of AP-1, to determine the target of genkwanin. MAPK signal transduction pathways are classified into three components. Hence, the effects of genkwanin on LPS-induced phosphorylation of p38, ERK1/2 and JNK were investigated. The results indicate that genkwanin suppresses the phosphorylation of p38 and JNK in a concentration-dependent manner, but little affects ERK1/2 phosphorylation (Figure 4A).

Originally identified as an immediate early gene, MKP-1 was then found to be a dual specificity phosphatase acting as a negative regulator of ERK1/2, JNK and p38 MAPK activities, with predominant effects on the latter two [31]–[36]. Thus, we next explored the effect of genkwanin on MKP-1. As shown in Figure 4B–C, LPS stimulation induced the expression of MKP-1 at the transcriptional and translational levels. Pretreatment with genkwanin markedly up-regulated the expression of MKP-1 without affecting MKP-1 mRNA.

### Genkwanin exerts anti-inflammatory effects mainly through decreasing miR-101 production

**Figure 5:**
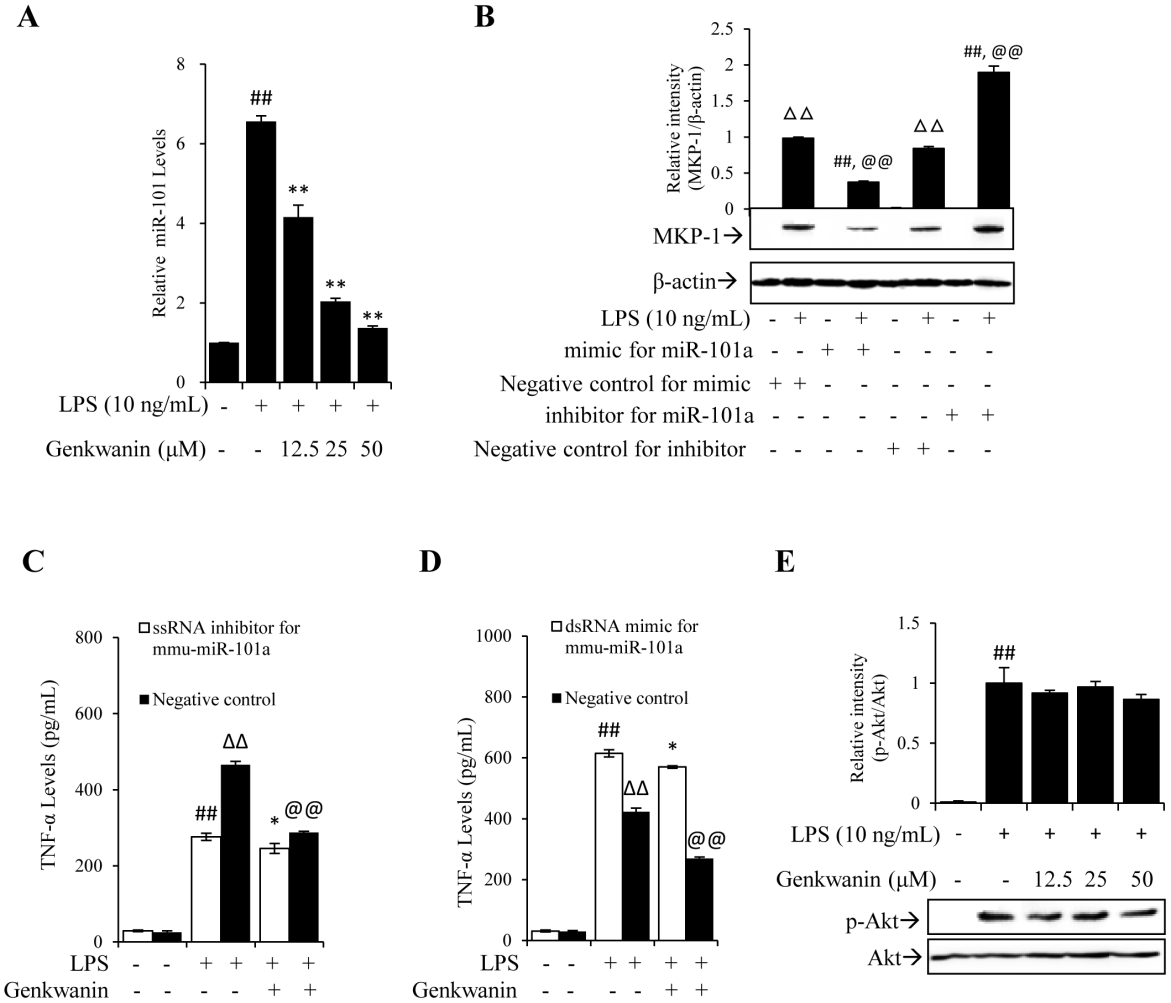
Genkwanin exerts anti-inflammatory effects mainly through decreasing miR-101 production. (A) Effect of genkwanin on miR-101 expression in LPS-activated RAW264.7 macrophages. The cells were pretreated with genkwanin at the indicated concentrations for 2 h and then exposed to LPS (10 ng/mL) for 90 min. miR-101 expression was determined by RT-*q*PCR analysis. (B) Effect of miR-101 on MKP-1 protein level. RAW264.7 macrophages were transfected with miR-101 mimic or inhibitor or their negative controls, and then stimulated with LPS (10 ng/mL) for 1 h. Cell lysates were analysed by Western blot. (C–D) Effects of genkwanin on supernatant TNF-α in LPS-stimulated RAW264.7 macrophages which have been transfected with ssRNA inhibitor (C) or dsRNA mimic (D) for mmu- miR-101a. RAW264.7 macrophages were transfected with miR-101 inhibitor or mimic or their negative controls, and then stimulated with LPS (10 ng/mL) for 24 h in the presence or absence of genkwanin (50 µM). Supernatant TNF-α was measured by ELISA. ^##^ *p*<0.01 vs. resting cells transfected with miR-101 inhibitor or mimic; *p<0.05 vs. LPS-treated cells transfected with miR-101 inhibitor or mimic; ΔΔ*p*<0.01 vs. resting cells transfected with negative controls; @@ *p*<0.01 vs. LPS-treated cells transfected with negative controls. (E) Effect of genkwanin on the LPS-induced p-Akt. Cells were pretreated with genkwanin at indicated concentrations for 2 h and then exposed to LPS (10 ng/mL) for 1 h. Cell lysated were assayed by Western blot analysis using Akt and p-Akt antibodies. ^##^ *p*<0.01 vs. normal control group. Bars represent mean ±SD of three independent experiments.

Our above results have demonstrated that genkwanin up-regulates MKP-1 at the posttranscriptional level. It was previously found that MKP-1 as a target of miR-101 which can repress MKP-1 protein expression [37]. Thus, we next evaluated the effect of genkwanin on miR-101 expression by RT-*q*PCR. As shown in Figure 5A, LPS induced the expression of miR-101, but pretreatment with genkwanin significantly decreased miR-101. We also analysed the effect of miR-101 on the protein level of MKP-1 in LPS-activated RAW264.7 macrophages which had been transfected with dsRNA mimic or ssRNA inhibitor for mmu-miR-101a (miR-101 mimic or miR-101 inhibitor). As shown in Figure 5B, transfection with miR-101 mimic significantly inhibited the production of MKP-1 protein, while miR-101 inhibitor markedly increased MKP-1 protein. These results are consistent with the previous reports that miR-101 is a negative regulator of MKP-1 expression [37].

To understand how genkwanin suppressed inflammation via miR-101, we transfected miR-101 inhibitor into RAW264.7 macrophages. In the resulting miR-101-deficient cells, the TNF-α production in response to LPS was significantly decreased as compared with the cells transfected with ssRNA negative control (NC) (Figure 5C). Genkwanin potently decreased LPS-induced supernatant TNF-α with an inhibition rate (IR) value of ∼38% {IR%  =  [(TNF-α _LPS_ - TNF-α _LPS+Genkwanin_)/TNF-α _LPS_] ×100} in ssRNA NC cells. However, in miR-101-deficient cells, genkwanin only slightly decreased LPS-induced supernatant TNF-α with an IR value of ∼10%, indicating that miR-101 played a predominant role in the anti-inflammatory activity of genkwanin.

Next, we transfected miR-101 mimic into RAW264.7 macrophages. In the resulting miR-101-abundant cells, the TNF-α production in response to LPS was significantly increased as compared with the cells transfected with dsRNA NC (Figure 5D). Genkwanin significantly decreased LPS-induced TNF-α with an IR value of ∼36% in dsRNA NC cells, while in miR-101-abundant cells, the IR value dropped to ∼7%, indicating that genkwanin was not effective against exogenous miR-101. Similar effects of miR-101 inhibitor and mimic on supernatant NO, IL-1β and IL-6 were also observed (Figure S2). Next, we evaluated the effects of genkwanin on the phosphorylation level of Akt in LPS-activated RAW264.7 macrophages. As shown in Figure 5E, LPS could induce the phosphorylation of Akt, but genkwanin did not appreciably affect the level of phospho-Akt (p-Akt).

## Discussion

**Figure 6:**
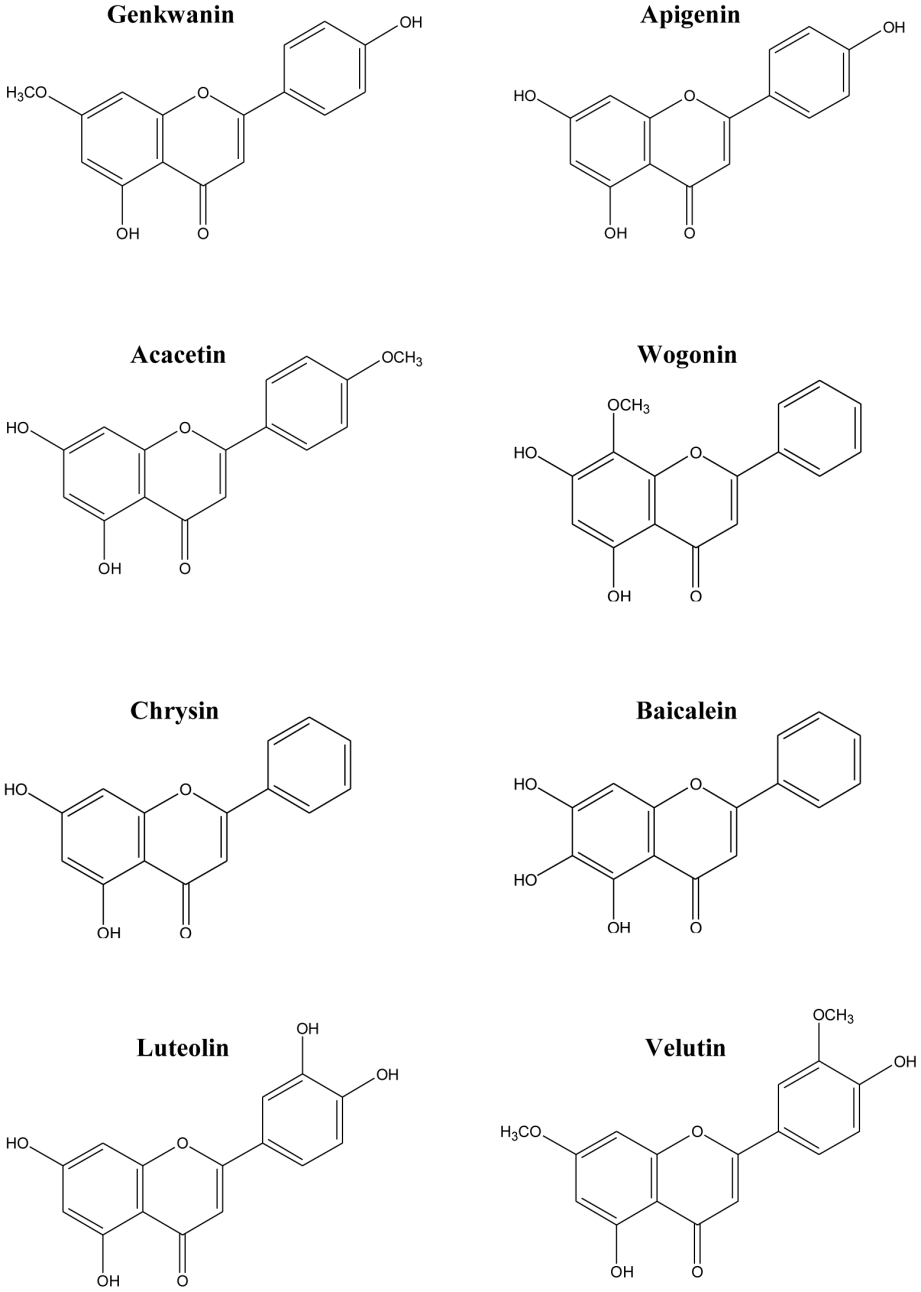
Chemical structures of genkwanin, apigenin, acacetin, wogonin, chrysin, baicalein, luteolin and velutin.

Genkwanin (4′,5-dihydroxy-7-methoxyflavone), as one of the major bioactive components in Genkwa Flos, is used as a representative index for the quality control of this herb and are included in the State Pharmacopoeia Commission of the People's Republic of China [1]. Many of its structurally similar analogues, such as apigenin [38], acacetin [39], chrysin [40], baicalein [41], wogonin [42], luteolin [43] and velutin [44] (Figure 6), were reported to suppress proinflammatory mediators in LPS-stimulated macrophages. Analysis of the structure-activity relationships of flavones showed that the anti-inflammatory effects could be enhanced by the methoxylation of the 5- or 7-hydroxyl groups on the A-ring or non-methoxylation of the 3′-hydroxyl groups on the B-ring [45]. Coincidentally, the chemical structure of genkwanin includes these dispositions, such as the 5-OCH_3_ and 3′-OH. Indeed, our results show that genkwanin (12.5 µM - 50 µM) potently decreases LPS-induced proinflammatory mediators, such as iNOS, TNF-α, IL-1β and IL-6 at the transcriptional and translational levels in RAW264.7 macrophages without cytotoxicity (Figures 1 and 2), indicating genkwanin's excellent anti-inflammatory potency.

Of course, due to the minor structural differences, the anti-inflammatory mechanisms of the analogues are diverse. For example, apigenin [46], baicalein [47] and luteolin [43] exert their anti-inflammatory effects mainly by inactivating NF-κB, while wogonin [48] can block JNK phosphorylation. Specially, acacetin [39] and velutin [44] can inactivate both NF-κB and MAPK. In this study, after carrying out Western blot assays by using phosphorylation antibodies, we found that genkwanin decreased the LPS-induced phospho-p38 (p-p38) and phospho-JNK (p-JNK) levels, but had no effect on phospho-ERK1/2 (p-ERK1/2) (Figure 4A).

Reversible activation MAPK requires phosphorylation on threonine and tyrosine residues of the activation domain of p38, JNK and ERK1/2. They are negatively regulated by a family of dual-specificity (threonine/tyrosine) phosphatases known as the MAPK phosphatases (MKPs) [49]. MKP-1, a stress-responsive MKP, localizes to the nucleus through its N terminus [50] and preferentially dephosphorylates activated p38 and JNK relative to ERK1/2. In LPS-stimulated mouse macrophages, MKP-1 shows a transient expression pattern with rapid induction, followed by a quick return to basal levels [51]. It is a critical negative regulator of macrophage signaling in response to inflammatory stimuli and is responsible for switching off the production of proinflammatory cytokines [51]–[53]. Therefore, the differential regulations of genkwanin on MAPK phosphorylation strongly suggest that the upstream regulator may be MKP-1. As expected, our results show that genkwanin increases the MKP-1 expression at the posttranscriptional level (Figure 4B–C).

MicroRNAs (miRNAs), the short (∼22 nucleotides) non-coding RNAs, play a central role in the regulation of gene expression at the posttranscriptional level via an RNA interference mechanism [54]. Recently, it was found that miR-101, a tumor-related miRNA, repress MKP-1 expression by binding to the 3′ untranslated region of MKP-1 in a direct and sequence-specific manner [37]. In our study, we also found the negative regulatory effect of miR-101 on MKP-1 protein (Figure 5B). Moreover, in response to LPS, the supernatant TNF-α, NO, IL-1β and IL-6 levels of miR-101 deficient cells is decreased (Figures 5C and S2), while these levels of miR-101-abundant cells is increased (Figures 5D and S2). Based on these slight effects of genkwanin on LPS-induced TNF-α, NO, IL-1β and IL-6 in exogenous miR-101-abundant cells (Figure 5D), we infer that genkwanin up-regulates MKP-1 protein may be attributed to the decrease of miR-101 production. In LPS-stimulated miR-101-deficient cells, genkwanin still somewhat suppresses supernatant TNF-α, NO, IL-1β and IL-6 (Figures 5C and S2), which suggests another mechanism, not depending on miR-101, remains possible. As predicted, genkwanin not only can decrease the phosphorylation level of JNK (Figure 4A), but also can directly inhibit the activity of p-JNK (Figure S3).

PI3K/Akt is known to regulate proinflammatory cytokine expression, but its exact role (positive versus negative) is controversial. Some studies have demonstrated that the PI3K/Akt pathway negatively regulates TLR-induced MAPK activation and proinflammatory cytokine production [55]–[58]. Other reports, however, have displayed that the PI3K/Akt implicated as a positive regulator of TLR-induced inflammatory response [59]–[61]. Zhu and his colleagues [37] proposed that PI3K/Akt negatively regulated the expression of MKP-1 through the induction of miR-101. However, our results indicated that genkwanin could not significantly affect the level of p-Akt (Figure 5E), suggesting that the phosphorylation of Akt may be not responsible for the effect of genkwanin on miR-101 production.

**Figure 7:**
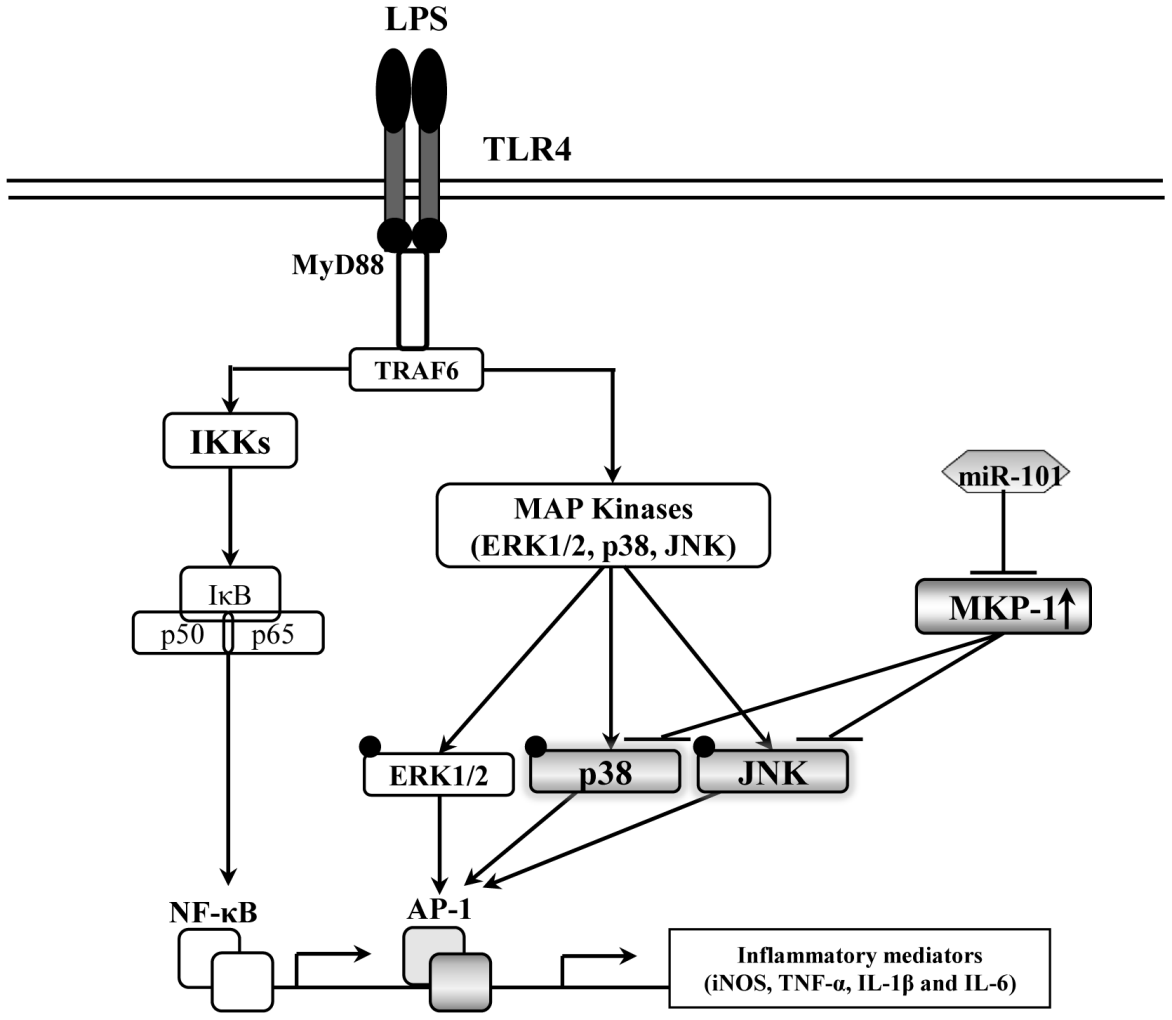
Proposed mechanism by which genkwanin exerts anti-inflammatory activity in LPS-activated RAW264.7 macrophages. The gray color indicates the targets of genkwanin.

Taken together, our results demonstrate that the anti-inflammatory effect of genkwanin may be mainly attributed to the down-regulation of the LPS-induced miR-101, thus increasing the protein expression of MKP-1, which dephosphorylates p38 and JNK in RAW264.7 macrophages (Figure 7). To our knowledge, genkwanin is the first compound derived from plant source shown to exert its anti-inflammatory activities mainly through the decrease of miR-101 production.

## Supporting Information

Figure S1 **Effect of genkwanin on cell viability.** RAW264.7 macrophages were incubated with genkwanin for 24 h and the cell viability were evaluated by MTT assay. Data represent the mean ± SD of three independent experiments.
(TIF)

Figure S2 **Effects of genkwanin on supernatant NO, IL-1β and IL-6 in LPS-stimulated RAW264.7 macrophages which have been transfected with ssRNA inhibitor or dsRNA mimic for mmu-miR-101a.** RAW264.7 macrophages were transfected with miR-101 inhibitor or mimic or their negative controls, and then stimulated with LPS (10 ng/mL) for 24 h in the presence or absence of genkwanin (50 µM). Supernatant NO (A–B), IL-1β (C–D) and IL-6 (E–F) were measured. ^##^ *p*<0.01 vs. resting cells transfected with miR-101 inhibitor or mimic; *p<0.05 vs. LPS-treated cells transfected with miR-101 inhibitor or mimic; ΔΔ*p*<0.01 vs. resting cells transfected with negative controls; @@ *p*<0.01 vs. LPS-treated cells transfected with negative controls.
(TIF)

Figure S3 **Effect of genkwanin on p-JNK activity.** RAW264.7 macrophages were treated with or without LPS (10 ng/mL) for 1 h. The intracellular p-JNK was extracted and purified by immunoprecipitation. The obtained p-JNK was treated with genkwanin for 15 min at room temperature and then incubated with c-Jun protein and ATP substrate. The effect of genkwanin on p-JNK activity was assayed by Western blot analysis and represented as the blots of p-c-Jun. All of the extraction and purification of p-JNK and the kinase activity assay were performed according to the manufacturer's instructions of KinaseSTAR JNK Activity Assay Kit (BioVision, Inc., San Francisco, California, USA). ^##^ *p*<0.01 vs. normal control group; **p<0.01 vs. LPS alone. Bars represent mean ±SD of three independent experiments.
(TIF)

## References

[1] The State Commission of Chinese Pharmacopoeia (2010) Pharmacopoeia of People's Republic of China. Part I. Beijing: Chemical Industry Press. p148.

[2] Altinier G, Sosa S, Aquino RP, Mencherini T, Della Loggia R, et al (2007) Characterization of topical antiinflammatory compounds in Rosmarinus officinalis L. J Agric Food Chem 55: 1718–1723. 17288440 10.1021/jf062610+

[3] Sadhu SK, Okuyama E, Fujimoto H, Ishibashi M, Yesilada E (2006) Prostaglandin inhibitory and antioxidant components of Cistus laurifolius, a Turkish medicinal plant. J Ethnopharmacol 108: 371–378. 16814498 10.1016/j.jep.2006.05.024

[4] Cottiglia F, Loy G, Garau D, Floris C, Casu M, et al (2001) Antimicrobial evaluation of coumarins and flavonoids from the stems of Daphne gnidium L. Phytomedicine 8: 302–305. 11515721 10.1078/0944-7113-00036

[5] Martini ND, Katerere DR, Eloff JN (2004) Biological activity of five antibacterial flavonoids from Combretum erythrophyllum (Combretaceae). J Ethnopharmacol 93: 207–212. 15234754 10.1016/j.jep.2004.02.030

[6] Kraft C, Jenett-Siems K, Siems K, Jakupovic J, Mavi S, et al (2003) In vitro antiplasmodial evaluation of medicinal plants from Zimbabwe. Phytother Res 17: 123–128. 12601673 10.1002/ptr.1066

[7] Kim AR, Zou YN, Park TH, Shim KH, Kim MS, et al (2004) Active components from Artemisia iwayomogi displaying ONOO(-) scavenging activity. Phytother Res 18: 1–7. 14750192 10.1002/ptr.1358

[8] Suh N, Luyengi L, Fong HH, Kinghorn AD, Pezzuto JM (1995) Discovery of natural product chemopreventive agents utilizing HL-60 cell differentiation as a model. Anticancer Res 15: 233–239. 7762989

[9] Brozic P, Kocbek P, Sova M, Kristl J, Martens S, et al (2009) Flavonoids and cinnamic acid derivatives as inhibitors of 17beta-hydroxysteroid dehydrogenase type 1. Mol Cell Endocrinol 301: 229–234. 18835421 10.1016/j.mce.2008.09.004

[10] Pelzer LE, Guardia T, Osvaldo Juarez A, Guerreiro E (1998) Acute and chronic antiinflammatory effects of plant flavonoids. Farmaco 53: 421–424. 9764475 10.1016/s0014-827x(98)00046-9

[11] Lin MW, Tsao LT, Chang LC, Chen YL, Huang LJ, et al (2007) Inhibition of lipopolysaccharide-stimulated NO production by a novel synthetic compound CYL-4d in RAW 264.7 macrophages involving the blockade of MEK4/JNK/AP-1 pathway. Biochem Pharmacol 73: 1796–1806. 17379190 10.1016/j.bcp.2007.02.009

[12] Baggiolini M (1998) Chemokines and leukocyte traffic. Nature 392: 565–568. 9560152 10.1038/33340

[13] Fontaine C, Rigamonti E, Nohara A, Gervois P, Teissier E, et al (2007) Liver X receptor activation potentiates the lipopolysaccharide response in human macrophages. Circ Res 101: 40–49. 17540978 10.1161/CIRCRESAHA.106.135814

[14] Reimer T, Brcic M, Schweizer M, Jungi TW (2008) poly(I:C) and LPS induce distinct IRF3 and NF-kappaB signaling during type-I IFN and TNF responses in human macrophages. J Leukoc Biol 83: 1249–1257. 18252870 10.1189/jlb.0607412

[15] Poltorak A, He X, Smirnova I, Liu MY, Van Huffel C, et al (1998) Defective LPS signaling in C3H/HeJ and C57BL/10ScCr mice: mutations in Tlr4 gene. Science 282: 2085–2088. 9851930 10.1126/science.282.5396.2085

[16] Nishida E, Gotoh Y (1993) The MAP kinase cascade is essential for diverse signal transduction pathways. Trends Biochem Sci 18: 128–131. 8388132 10.1016/0968-0004(93)90019-j

[17] Su B, Karin M (1996) Mitogen-activated protein kinase cascades and regulation of gene expression. Curr Opin Immunol 8: 402–411. 8793994 10.1016/s0952-7915(96)80131-2

[18] Dong C, Davis RJ, Flavell RA (2002) MAP kinases in the immune response. Annu Rev Immunol 20: 55–72. 11861597 10.1146/annurev.immunol.20.091301.131133

[19] Zarubin T, Han J (2005) Activation and signaling of the p38 MAP kinase pathway. Cell Res 5: 11–18. 10.1038/sj.cr.7290257 15686620

[20] Chen YC, Shen SC, Lee WR, Hou WC, Yang LL, et al (2001) Inhibition of nitric oxide synthase inhibitors and lipopolysaccharide induced inducible NOS and cyclooxygenase-2 gene expressions by rutin, quercetin, and quercetin pentaacetate in RAW 264.7 macrophages. J Cell Biochem 82: 537–548. 11500931 10.1002/jcb.1184

[21] Li M, Zhang L, Cai RL, Gao Y, Qi Y (2012) Lipid-soluble extracts from Salvia miltiorrhiza inhibit production of LPS-induced inflammatory mediators via NF-kappaB modulation in RAW 264.7 cells and perform antiinflammatory effects in vivo. Phytother Res 26: 1195–1204. 22228586 10.1002/ptr.3680

[22] Pinho BR, Sousa C, Valentão P, Andrade PB (2011) Is nitric oxide decrease observed with naphthoquinones in LPS stimulated RAW 264.7 macrophages a beneficial property? PloS One 6: e24098. 21887376 10.1371/journal.pone.0024098 PMC3162593

[23] Ochoa JB, Udekwu AO, Billiar TR, Curran RD, Cerra FB, et al (1991) Nitrogen oxide levels in patients after trauma and during sepsis. Ann Surg 214: 621–626. 1953116 10.1097/00000658-199111000-00013 PMC1358619

[24] MacMicking JD, Nathan C, Hom G, Chartrain N, Fletcher DS, et al (1995) Altered responses to bacterial infection and endotoxic shock in mice lacking inducible nitric oxide synthase. Cell 81: 641–650. 7538909 10.1016/0092-8674(95)90085-3

[25] Wang QZ, Jacobs J, DeLeo J, Kruszyna H, Kruszyna R, et al (1991) Nitric oxide hemoglobin in mice and rats in endotoxic shock. Life Sci 49: PL55–PL60. 1652047 10.1016/0024-3205(91)90251-6

[26] Murphy S (2000) Production of nitric oxide by glial cells: regulation and potential roles in the CNS. Glia 29: 1–13. 10594918 10.1002/(sici)1098-1136(20000101)29:1<1::aid-glia1>3.0.co;2-n

[27] Vaillancourt F, Morquette B, Shi Q, Fahmi H, Lavigne P, et al (2007) Differential regulation of cyclooxygenase-2 and inducible nitric oxide synthase by 4-hydroxynonenal in human osteoarthritic chondrocytes through ATF-2/CREB-1 transactivation and concomitant inhibition of NF-kappaB signaling cascade. J Cell Biochem 100: 1217–1231. 17031850 10.1002/jcb.21110

[28] Chung HJ, Lee HS, Shin JS, Lee SH, Park BM, et al (2010) Modulation of acute and chronic inflammatory processes by a traditional medicine preparation GCSB-5 both in vitro and in vivo animal models. J Ethnopharmacol 130: 450–459. 20621661 10.1016/j.jep.2010.05.020

[29] Choi Y, Lee MK, Lim SY, Sung SH, Kim YC (2009) Inhibition of inducible NO synthase, cyclooxygenase-2 and interleukin-1beta by torilin is mediated by mitogen-activated protein kinases in microglial BV2 cells. Br J Pharmacol 156: 933–940. 19298258 10.1111/j.1476-5381.2009.00022.x PMC2697713

[30] Ajmone-Cat MA, De Simone R, Nicolini A, Minghetti L (2003) Effects of phosphatidylserine on p38 mitogen activated protein kinase, cyclic AMP responding element binding protein and nuclear factor-kappaB activation in resting and activated microglial cells. J Neurochem 84: 413–416. 12559004 10.1046/j.1471-4159.2003.01562.x

[31] Camps M, Nichols A, Arkinstall S (2000) Dual specificity phosphatases: a gene family for control of MAP kinase function. FASEB J 14: 6–16. 10627275

[32] Karin M (2004) Mitogen activated protein kinases as targets for development of novel anti-inflammatory drugs. Ann Rheum Dis 63 (Suppl 2): ii62–ii64. 10.1136/ard.2004.028274 PMC1766783 15479874

[33] Theodosiou A, Ashworth A (2002) MAP kinase phosphatases. Genome Biol 3: REVIEWS3009. 12184814 10.1186/gb-2002-3-7-reviews3009 PMC139386

[34] Charles CH, Abler AS, Lau LF (1992) cDNA sequence of a growth factor-inducible immediate early gene and characterization of its encoded protein. Oncogene 7: 187–190. 1741163

[35] Charles CH, Sun H, Lau LF, Tonks NK (1993) The growth factor-inducible immediate-early gene 3CH134 encodes a protein-tyrosine-phosphatase. Proc Natl Acad Sci U S A 90: 5292–5296. 8389479 10.1073/pnas.90.11.5292 PMC46702

[36] Sun H, Charles CH, Lau LF, Tonks NK (1993) MKP-1 (3CH134), an immediate early gene product, is a dual specificity phosphatase that dephosphorylates MAP kinase in vivo. Cell 75: 487–493. 8221888 10.1016/0092-8674(93)90383-2

[37] Zhu QY, Liu Q, Chen JX, Lan K, Ge BX (2010) MicroRNA-101 targets MAPK phosphatase-1 to regulate the activation of MAPKs in macrophages. J Immunol 185: 7435–7442. 21068409 10.4049/jimmunol.1000798

[38] Smolinski AT, Pestka JJ (2003) Modulation of lipopolysaccharide-induced proinflammatory cytokine production in vitro and in vivo by the herbal constituents apigenin (chamomile), ginsenoside Rb(1) (ginseng) and parthenolide (feverfew). Food Chem Toxicol 41: 1381–1390. 12909272 10.1016/s0278-6915(03)00146-7

[39] Pan MH, Lai CS, Wang YJ, Ho CT (2006) Acacetin suppressed LPS-induced up-expression of iNOS and COX-2 in murine macrophages and TPA-induced tumor promotion in mice. Biochem Pharmacol 72: 1293–1303. 16949556 10.1016/j.bcp.2006.07.039

[40] Harasstani OA, Moin S, Tham CL, Liew CY, Ismail N, et al (2010) Flavonoid combinations cause synergistic inhibition of proinflammatory mediator secretion from lipopolysaccharide-induced RAW 264.7 cells. Inflamm Res 59: 711–721. 20221843 10.1007/s00011-010-0182-8

[41] Oh YC, Cho WK, Jeong YH, Im GY, Lee KJ, et al (2013) Anti-inflammatory effect of Sosihotang via inhibition of nuclear factor-kappaB and mitogen-activated protein kinases signaling pathways in lipopolysaccharide-stimulated RAW 264.7 macrophage cells. Food Chem Toxicol 53: 343–351. 23246826 10.1016/j.fct.2012.12.006

[42] Kaneko T, Chiba H, Horie N, Kato T, Kobayashi M, et al (2009) Effect of Scutellariae radix ingredients on prostaglandin E(2) production and COX-2 expression by LPS-activated macrophage. In Vivo 23: 577–581. 19567393

[43] Park CM, Jin KS, Lee YW, Song YS (2011) Luteolin and chicoric acid synergistically inhibited inflammatory responses via inactivation of PI3K-Akt pathway and impairment of NF-kappaB translocation in LPS stimulated RAW 264.7 cells. Eur J Pharmacol 660: 454–459. 21513709 10.1016/j.ejphar.2011.04.007

[44] Xie C, Kang J, Li Z, Schauss AG, Badger TM, et al (2012) The acai flavonoid velutin is a potent anti-inflammatory agent: blockade of LPS-mediated TNF-alpha and IL-6 production through inhibiting NF-kappaB activation and MAPK pathway. J Nutr Biochem 23: 1184–1191. 22137267 10.1016/j.jnutbio.2011.06.013

[45] During A, Larondelle Y (2013) The O-methylation of chrysin markedly improves its intestinal anti-inflammatory properties: Structure-activity relationships of flavones. Biochem Pharmacol 86: 1739–1746. 24134915 10.1016/j.bcp.2013.10.003

[46] Nicholas C, Batra S, Vargo MA, Voss OH, Gavrilin MA, et al (2007) Apigenin blocks lipopolysaccharide-induced lethality in vivo and proinflammatory cytokines expression by inactivating NF-kappaB through the suppression of p65 phosphorylation. J Immunol 179: 7121–7127. 17982104 10.4049/jimmunol.179.10.7121

[47] Fan GW, Zhang Y, Jiang X, Zhu Y, Wang B, et al (2013) Anti-inflammatory Activity of Baicalein in LPS-Stimulated RAW264.7 Macrophages via Estrogen Receptor and NF-kappaB-Dependent Pathways. Inflammation 36: 1584–1591. 23892998 10.1007/s10753-013-9703-2

[48] Huang GC, Chow JM, Shen SC, Yang LY, Lin CW, et al (2007) Wogonin but not Nor-wogonin inhibits lipopolysaccharide and lipoteichoic acid-induced iNOS gene expression and NO production in macrophages. Int Immunopharmacol 7: 1054–1063. 17570322 10.1016/j.intimp.2007.04.001

[49] Carlson J, Cui W, Zhang Q, Xu X, Mercan F, et al (2009) Role of MKP-1 in osteoclasts and bone homeostasis. Am J Pathol 175: 1564–1573. 19762714 10.2353/ajpath.2009.090035 PMC2751553

[50] Wu JJ, Zhang L, Bennett AM (2005) The noncatalytic amino terminus of mitogen-activated protein kinase phosphatase 1 directs nuclear targeting and serum response element transcriptional regulation. Mol Cell Biol 25: 4792–4803. 15899879 10.1128/MCB.25.11.4792-4803.2005 PMC1140620

[51] Chi H, Barry SP, Roth RJ, Wu JJ, Jones EA, et al (2006) Dynamic regulation of pro- and anti-inflammatory cytokines by MAPK phosphatase 1 (MKP-1) in innate immune responses. Proc Natl Acad Sci U S A 103: 2274–2279. 16461893 10.1073/pnas.0510965103 PMC1413743

[52] Chen P, Li J, Barnes J, Kokkonen GC, Lee JC, et al (2002) Restraint of proinflammatory cytokine biosynthesis by mitogen-activated protein kinase phosphatase-1 in lipopolysaccharide-stimulated macrophages. J Immunol 169: 6408–6416. 12444149 10.4049/jimmunol.169.11.6408

[53] Rodriguez N, Dietrich H, Mossbrugger I, Weintz G, Scheller J, et al (2010) Increased inflammation and impaired resistance to Chlamydophila pneumoniae infection in Dusp1(-/-) mice: critical role of IL-6. J Leukoc Biol 88: 579–87. 20483921 10.1189/jlb.0210083

[54] Ambros V (2004) The functions of animal microRNAs. Nature 431: 350–355. 15372042 10.1038/nature02871

[55] Guha M, Mackman N (2002) The phosphatidylinositol 3-kinase-Akt pathway limits lipopolysaccharide activation of signaling pathways and expression of inflammatory mediators in human monocytic cells. J Biol Chem 277: 32124–32132. 12052830 10.1074/jbc.M203298200

[56] Fukao T, Koyasu S (2003) PI3K and negative regulation of TLR signaling. Trends Immunol 24: 358–363. 12860525 10.1016/s1471-4906(03)00139-x

[57] Yu Y, Nagai S, Wu H, Neish AS, Koyasu S, et al (2006) TLR5-mediated phosphoinositide 3-kinase activation negatively regulates flagellin-induced proinflammatory gene expression. J Immunol 176: 6194–6201. 16670329 10.4049/jimmunol.176.10.6194

[58] Medina EA, Morris IR, Berton MT (2010) Phosphatidylinositol 3-kinase activation attenuates the TLR2-mediated macrophage proinflammatory cytokine response to Francisella tularensis live vaccine strain. J Immunol 185: 7562–7572. 21098227 10.4049/jimmunol.0903790

[59] Ojaniemi M, Glumoff V, Harju K, Liljeroos M, Vuori K, et al (2003) Phosphatidylinositol 3-kinase is involved in Toll-like receptor 4-mediated cytokine expression in mouse macrophages. Eur J Immunol 33: 597–605. 12616480 10.1002/eji.200323376

[60] Lee JY, Ye J, Gao Z, Youn HS, Lee WH, et al (2003) Reciprocal modulation of Toll-like receptor-4 signaling pathways involving MyD88 and phosphatidylinositol 3-kinase/AKT by saturated and polyunsaturated fatty acids. J Biol Chem 278: 37041–37051. 12865424 10.1074/jbc.M305213200

[61] Rajaram MV, Ganesan LP, Parsa KV, Butchar JP, Gunn JS, et al (2006) Akt/Protein kinase B modulates macrophage inflammatory response to Francisella infection and confers a survival advantage in mice. J Immunol 177: 6317–6324. 17056562 10.4049/jimmunol.177.9.6317

