# Supplementary figures and images for: Genkwanin Inhibits Proinflammatory Mediators Mainly through the Regulation of miR-101/MKP-1/MAPK Pathway in LPS-Activated Macrophages
Source: PLoS One. 2014 May 6;9(5):e96741. doi: 10.1371/journal.pone.0096741 (PMC4011752; doi:10.1371/journal.pone.0096741)

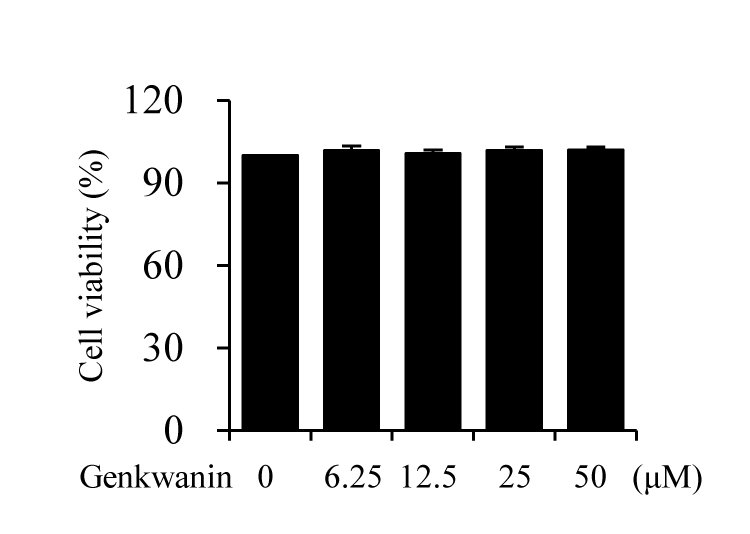

Supplement: Figure S1 — Effect of genkwanin on cell viability. RAW264.7 macrophages were incubated with genkwanin for 24 h and the cell viability were evaluated by MTT assay. Data represent the mean ± SD of three independent experiments. (TIF) [file pone.0096741.s001.tif]

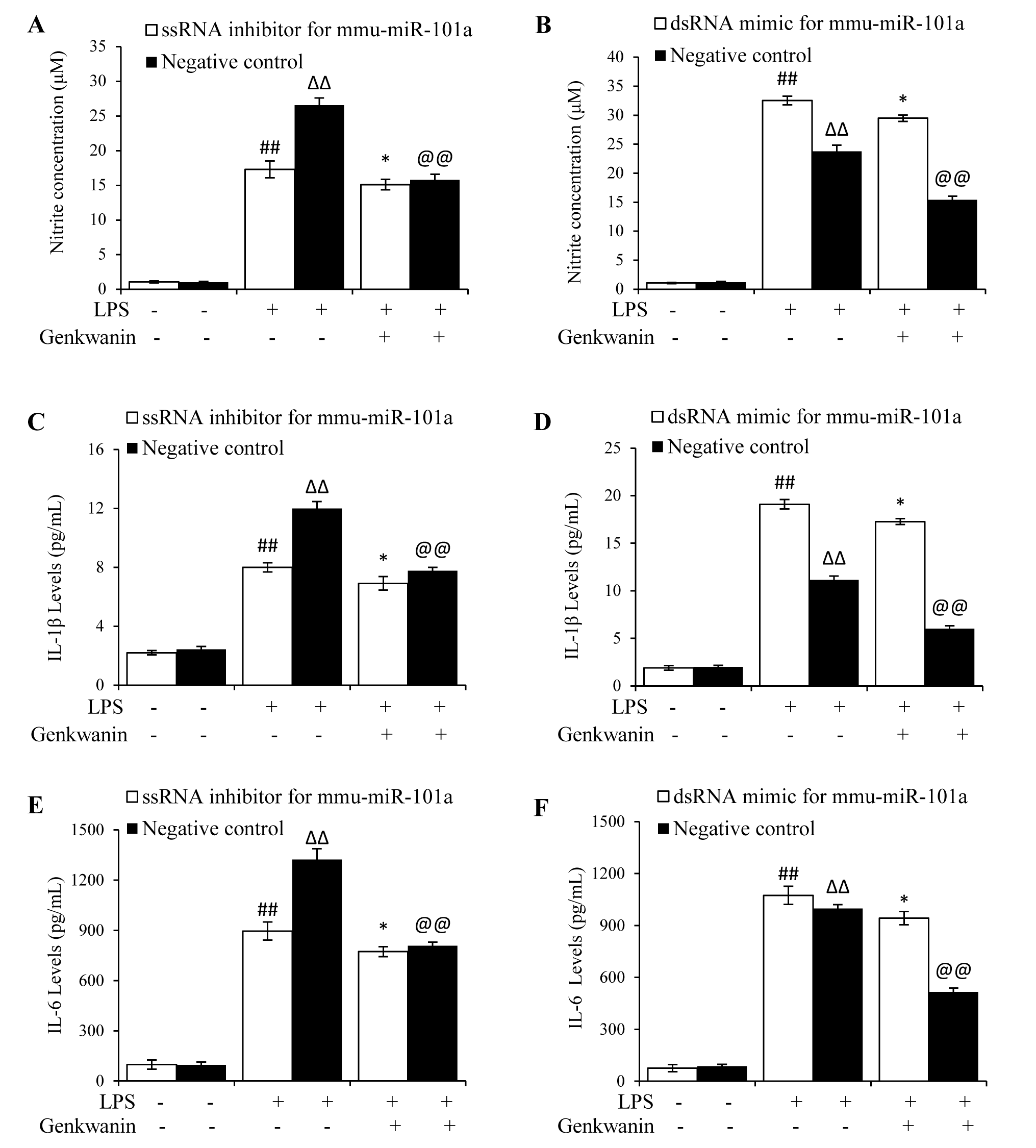

Supplement: Figure S2 — Effects of genkwanin on supernatant NO, IL-1β and IL-6 in LPS-stimulated RAW264.7 macrophages which have been transfected with ssRNA inhibitor or dsRNA mimic for mmu-miR-101a. RAW264.7 macrophages were transfected with miR-101 inhibitor or mimic or their negative controls, and then stimulated with LPS (10 ng/mL) for 24 h in the presence or absence of genkwanin (50 µM). Supernatant NO (A–B), IL-1β (C–D) and IL-6 (E–F) were measured. ## p<0.01 vs. resting cells transfected with miR-101 inhibitor or mimic; *p<0.05 vs. LPS-treated cells transfected with miR-101 inhibitor or mimic; ΔΔp<0.01 vs. resting cells transfected with negative controls; @@ p<0.01 vs. LPS-treated cells transfected with negative controls. (TIF) [file pone.0096741.s002.tif]

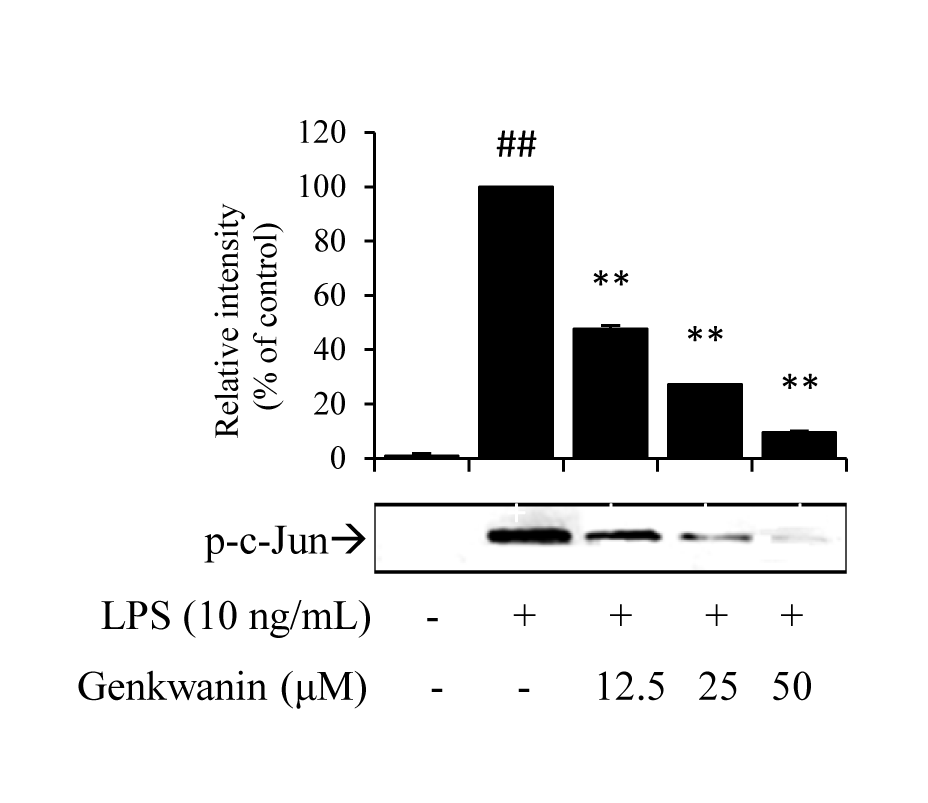

Supplement: Figure S3 — Effect of genkwanin on p-JNK activity. RAW264.7 macrophages were treated with or without LPS (10 ng/mL) for 1 h. The intracellular p-JNK was extracted and purified by immunoprecipitation. The obtained p-JNK was treated with genkwanin for 15 min at room temperature and then incubated with c-Jun protein and ATP substrate. The effect of genkwanin on p-JNK activity was assayed by Western blot analysis and represented as the blots of p-c-Jun. All of the extraction and purification of p-JNK and the kinase activity assay were performed according to the manufacturer's instructions of KinaseSTAR JNK Activity Assay Kit (BioVision, Inc., San Francisco, California, USA). ## p<0.01 vs. normal control group; **p<0.01 vs. LPS alone. Bars represent mean ±SD of three independent experiments. (TIF) [file pone.0096741.s003.tif]
